# RGAugury: a pipeline for genome-wide prediction of resistance gene analogs (RGAs) in plants

**DOI:** 10.1186/s12864-016-3197-x

**Published:** 2016-11-02

**Authors:** Pingchuan Li, Xiande Quan, Gaofeng Jia, Jin Xiao, Sylvie Cloutier, Frank M. You

**Affiliations:** 1 Morden Research and Development Centre, Agriculture and Agri-Food Canada, Morden, MB R6M 1Y5 Canada; 2 University of Saskatchewan, Saskatoon, SK S7N 5A8 Canada; 3 National Key Laboratory of Crop Genetics and Germplasm Enhancement, Cytogenetics Institute, Nanjing Agricultural University, Nanjing, 210095 China; 4 Ottawa Research and Development Centre, Agriculture and Agri-Food Canada, Ottawa, ON K1A 0C6 Canada

**Keywords:** Resistance gene analog (RGA), Nucleotide binding site (NBS), Receptor like protein (RLP), Receptor like kinase (RLK), Genome-wide prediction, Pipeline

## Abstract

**Background:**

Resistance gene analogs (RGAs), such as NBS-encoding proteins, receptor-like protein kinases (RLKs) and receptor-like proteins (RLPs), are potential *R*-genes that contain specific conserved domains and motifs. Thus, RGAs can be predicted based on their conserved structural features using bioinformatics tools. Computer programs have been developed for the identification of individual domains and motifs from the protein sequences of RGAs but none offer a systematic assessment of the different types of RGAs. A user-friendly and efficient pipeline is needed for large-scale genome-wide RGA predictions of the growing number of sequenced plant genomes.

**Results:**

An integrative pipeline, named RGAugury, was developed to automate RGA prediction. The pipeline first identifies RGA-related protein domains and motifs, namely nucleotide binding site (NB-ARC), leucine rich repeat (LRR), transmembrane (TM), serine/threonine and tyrosine kinase (STTK), lysin motif (LysM), coiled-coil (CC) and Toll/Interleukin-1 receptor (TIR). RGA candidates are identified and classified into four major families based on the presence of combinations of these RGA domains and motifs: NBS-encoding, TM-CC, and membrane associated RLP and RLK. All time-consuming analyses of the pipeline are paralleled to improve performance. The pipeline was evaluated using the well-annotated Arabidopsis genome. A total of 98.5, 85.2, and 100 % of the reported NBS-encoding genes, membrane associated RLPs and RLKs were validated, respectively. The pipeline was also successfully applied to predict RGAs for 50 sequenced plant genomes. A user-friendly web interface was implemented to ease command line operations, facilitate visualization and simplify result management for multiple datasets.

**Conclusions:**

RGAugury is an efficiently integrative bioinformatics tool for large scale genome-wide identification of RGAs. It is freely available at Bitbucket: https://bitbucket.org/yaanlpc/rgaugury.

**Electronic supplementary material:**

The online version of this article (doi:10.1186/s12864-016-3197-x) contains supplementary material, which is available to authorized users.

## Background

Aside from physical and chemical barriers, plants protect themselves from pathogen infections by employing a sophisticated biochemical immune system composed mainly of two layers. The first layer is conferred by cell surface pattern-recognition receptors (PRRs) that detect general elicitors pathogen/microbe-associated molecule patterns (PAMP/MAMPs), known as PAMP-triggered immunity (PTI). The defence could be overcome by specific pathogen effectors [1, 2]. Plants have also evolved other types of receptors called resistance (*R*) proteins that recognize specific effectors and elicit a robust counter-attack system termed effector-triggered immunity (ETI) [3]. This second response layer corresponds to a gene-for-gene interaction [4].

Resistance gene analogs (RGAs) comprise both PRRs and *R*-genes and, most have conserved domains and motifs [5]. The majority of characterized PRRs are either surface-localized receptor-like protein kinases (RLKs) or membrane associated receptor-like proteins (RLPs) [6–8]. RLKs possess an extracellular sensing domain, a transmembrane (TM) region and an intracellular protein kinase, containing two types according to the domain structure, leucine rich repeat (LRR) type, such as FLS2 [9], EFR [10] and XA21 [11], and, lysin motif (LysM)-type, such as CERK1 [12]. RLPs have similar domain architecture to RLKs except for the absence of a kinase domain in their intracellular region [13], such as Cf-9 (LRR-type) [14], Eix1 and Eix2 (LRR-type) [15], CEBiP (LysM-type) [16]. R-proteins or effector-recognition receptors are described as intracellular immune receptors and most belong to nucleotide-binding site-LRR (NBS-LRR or NLR) class [17]. Seven domains or motifs may be found in R-proteins: Toll/Interleukin-1 receptor (TIR), coiled-coil (CC), leucine zipper (LZ), NBS, LRR, TM and serine-threonine kinase (STK) [18]. Based on these domains, R-proteins are categorized into five main classes: (1) CC-NBS-LRR (CNL), (2) TIR-NBS-LRR (TNL), (3) RLKs, (4) RLPs and (5) other variants [19].

Pyramiding of plant resistance genes in new cultivars is the most effective and environmentally friendly approach for plant disease control and reduction of yield losses. Development of diagnostic molecular markers associated with disease resistance is a prerequisite for molecular resistance breeding. Marker saturation in the vicinity of the target resistance gene is a critical step for mapped-based or positional cloning of R-genes which results in the development of diagnostic markers [20]. RGAs-based marker development strategies have been successfully applied for the development of diagnostic markers for orange wheat blossom midge and wheat stem rust resistance genes [21, 22]. This strategy involves four iterative steps: (1) identification of genome-wide RGAs, (2) identification of potential RGA candidates in the vicinity of the target resistance gene using comparative genomics analysis, (3) design of SNP markers for candidate RGAs, and (4) marker evaluation using biparental genetic populations and/or association panels. Therefore, the identification of genome-wide RGAs is a useful genomic resource for fine-mapping and cloning of resistance genes and, for marker development for resistance breeding.

To date, hundreds of NBS-LRR, RLK and RLP genes have been reported in plants [13, 23–27]. In these reports, RGAs were detected using several individual computing programs to predict related domains and/or motifs followed by manual or semi-automated summarization of the results with custom scripts. These programs include BLAST [28], Hmmer3 [29], InterProScan5 with Pfam and SMART databases [30, 31], Phobius [32], TMHMM [33], pfam_scan [34], and nCoils [35]. The diversity of tools and parameters used in these studies makes it impossible to properly compare their outputs [5]. In addition, tools, such as nCoils, Phobius and pfam_scan, do not have multiple-threading features or are not optimized for large datasets, limiting their performance in genome-wide analyses. As the number of sequenced genomes increase, rapid and accurate RGA identification will benefit genome annotation endeavours. Here, we developed a comprehensive pipeline to address these shortfalls. Our primary objective was to develop an efficient and integrative pipeline tool to identify all known types of RGA candidates from genome-scale datasets, with a user-friendly interface and, that would be fully integrated from dataset to summarization. The pipeline, which we named RGAugury, was validated using the well-annotated Arabidopsis genome data and successfully applied to 50 sequenced genomes for comparative analysis.

## Implementation

### Pipeline design

**Fig. 1:**
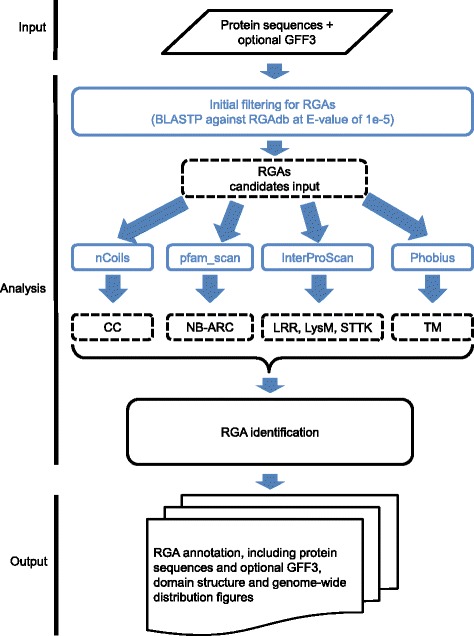
Workflow of RGAugury. The pipeline was designed to use protein sequences to detect conserved domains and motifs found in genes involved in plant resistance and identify RGAs by integrating results generated from five programs: BLAST, InterProScan, pfam_scan, nCoil and Phobius. The annotated candidates for four different RGA types are exported as plain files. Analyses performed in parallel mode are labelled in blue. Intermediate results are indicated by a dashed-line box. GFF3: Generic Feature Format version 3; CC: coiled-coil; LRR: leucine-rich repeat; NB-ARC: nucleotide binding adapter shared by APAF-1, R gene products and CED-4; STTK: serine/threonine and tyrosine kinase; LysM: lysin motif; TM: transmembrane

NBS and TM-CC containing proteins and membrane associated RLPs and RLKs belong to the main four known RGA families [5], and were thus included in the current version of RGAugury. RGA identification generally includes two basic steps: the identification of all conserved domains and/or motifs from protein sequences translated from gene sequences, followed by the analysis of the domain and/or motif composition including the classification of the genes into one of the four known types of RGAs based on such domain and/or motif structures. To improve the computing performance for the genome-wide identification of RGAs, three additional solutions were implemented in the pipeline: (1) an initial filtering of RGA candidates to remove a large portion of non-RGA genes, (2) parallel computing for time-consuming calculations, and (3) selection of the most efficient protein databases for domain detection. The design and the workflow of the pipeline are summarized in Fig. 1 and described in the following sections.

### Initial filtering of RGA candidates

Because RGAs occupies a small percentage of the total genes in a genome, initial filtering to remove non-RGA genes can dramatically reduce the number of genes for the downstream domain/motif detection which saves considerable computing time. To do so, we used BLASTP to identify potential RGA candidates against an RGA database called RGAdb (see description below). The BLAST+ package was selected for sequence alignment as it outperforms BLAST in calculation power under the same conditions [36]. In the initial step, the input protein sequences are aligned against RGAdb by BLASTP using an E-value cut-off of 1e-5 (this E-value cut-off may be adjusted according to different species). Non-RGA proteins are filtered out (Fig. 1). Based on the analyses of 50 sequenced plant genomes, an average of 76.4 % of the annotated genes were removed, significantly abridging the downstream analysis time.

RGAdb was constructed using protein sequences and their annotations from the NCBI non-redundant protein database (*nr*) (http://www.ncbi.nlm.nih.gov/), plant resistance gene database (PRGdb, http://www.prgdb.org) [19]) and other cloned *R*-genes. Sequence entries were retrieved from *nr* with the key words ‘resistance’ and ‘disease’. Disease-irrelevant entries such as resistance to aluminum, drought, cold, DNA-damage, herbicide and UVB were removed. A total of 14,906 disease resistance related sequences were retained. In addition, ‘contributed’, ‘putative’ and ‘references’ entries from PRGdb were merged and the resulting dataset was filtered to remove redundant and non-annotated entries. As a result, 44,109 entries from PRGdb were appended into RGAdb. A few NBS encoding proteins from wheat and Brachypodium were also added into RGAdb [37–39]. Finally RGAdb contains 59,597 entries which were derived from more than 300 plant species (Additional file 1) and 80 % of the database entries are NBS coding proteins, RLKs, RLPs, MLO- and RPW-like proteins. In addition, this database will be regularly maintained to remove irrelevant entries and add new RGA entries.

### Domain and motif detection

The initial filtering results in a reduced set of potential RGA candidates for the downstream domain and motif detection. There are seven RGAs-related domains and motifs, including NB-ARC or NBS, LRR, TM, STTK, LysM, CC and TIR in proteins. To detect them, four third-party tools were chosen and integrated into the pipeline. nCoils program was used to identify the CC domain present in CNL, CN and TM-CC types [35]. Minor modifications on the original source code of nCoils were made under its redistribution permission to facilitate the RGAugury pipeline to call nCoils through command-line. For detection of the NB-ARC domain, the pfam_scan toolkit was preferred because it outperformed InterProScan according to the test results obtained using the Arabidopsis genome data (data not shown). In addition, unlike InterProScan, the *P*-value parameter in pfam_scan is adjustable offering flexibility for the adjustment of the *P*-value cut-off across plant species. However, InterProScan was chosen to identify LRR and LysM, which are two components of RLPs and RLKs that play a role in pathogen signal recognition [16]. InterProScan is also suitable for the detection of the STTK domains of RLKs [25, 40–42]. Two tools, Phobius [32] and TMHMM [33], are available for TM domain identification but Phobius was elected because it performed better than TMHMM [32]. RGAugury’s configuration file can be easily modified to add any additional domains or motifs for detection, thus offering flexibility and extendibility.

InterProScan is a protein domain identifier that uses up to 14 databases for detection [43]. The accuracy and computing performance depend on the number and nature of the databases used for analysis. Pfam, Gene3D, SMART, Superfamily and the external database Panther were chosen for RGA domain detection based on accuracy and computing performance for the detection of LRR, LysM and STTK domains/motifs (data not shown). Three modes for database selection are provided in the pipeline. The default ‘Quick’ mode uses only Pfam and Gene3D which can identify most domains and motifs from input protein sequences. In the ‘Deep’ mode, Superfamily and SMART are also included for domain detection. As a consequence of the additional databases, the ‘Deep’ mode may perform slightly more slowly but this reduction in performance must be weighed against the accuracy and completeness of the results coveted. Differences between the two modes were mainly in the numbers of identified proteins with LRR motifs. The third mode is called ‘Free’ mode. This mode allows users to select one or more databases from a list. This mode is usually intended for result confirmation but can also be of use in custom applications. Here, the Panther (Protein Analysis Though Evolutionary Relationships) [44] database is introduced to the pipeline. Panther is a large protein database for comprehensive protein evolutionary and functional classification that contains more than 12,000 protein families from 104 sequenced genomes [45]. Our test results indicated that Panther would require huge calculation resource upon CPU threads for genome-scale protein analysis, which may take days to weeks depending on the input sequences. Thus, it is not suitable for genome-scale RGA identification but it can be accessed in the ‘Free’ mode for specific purposes such as the confirmation of small numbers of identified RGA candidates for example.

### RGA identification

**Fig. 2:**
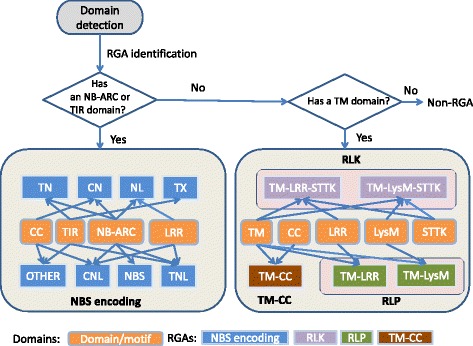
RGA identification based on domain structures of genes. CC: coiled-coil; CN: CC-NBS; CNL: CC-NBS-LRR; LRR: leucine rich repeat; LysM: lysin motif; NB-ARC: nucleotide binding site-activity regulated cytoskeleton; NBS: nucleotide-binding site; NL: NBS-LRR; RGA: resistance gene analog; RLK: receptor like kinase; RLP: receptor like protein; STTK: serine/threonine and tyrosine kinase; TIR: Toll/Interleukin-1 receptor; TM: transmembrane; TN: TIR-NBS; TNL: TIR-NBS-LRR; TX: TIR-unknown domain

Once all domains and motifs are identified from the input protein sequences, RGAugury creates a table that lists the RGA candidates initially identified by BLASTP, along with their identified domains and coordinates on genes. The RGA identification module was developed to classify genes as potential RGAs and to classify them into specific RGA families (Fig. 2). First, the program classifies genes containing an NB-ARC or a TIR domain into the NBS encoding family. Genes that contain a TM domain may belong to the RLP, RLK or TM-CC families depending on the presence of other domains in the gene structures. If no NB-ARC, TIR or TM is observed, the gene is discarded as a non-RGA. The NBS-encoding gene family members are further divided into several subgroups according to their domain architecture, namely NBS, CNL, TNL, TN, CN, NL, TX and OTHER that may have chimeric domain/motif architecture. Here C, N, L, T and X represent CC, NBS or NB-ARC, LRR, TIR and unknown domains, respectively. For example, a gene with CC, NB-ARC and LRR domains is classified in the CNL subgroup. However, a gene with both TIR and CC domains ends up in the OTHER subgroup. In this case, a user needs to manually check this unexpected domain combination using the exported domain information. A gene with only an NB-ARC domain would require confirmation by InterProScan.

### Data input and result output

The command-line pipeline requires three parameters. The first parameter is a protein sequence file in FASTA format from either a whole genome annotation project or manually annotated protein sequence data. The second one is the *P*-value cut-off for the initial RGA filtering using BLASTP. The default value is set to 1e-5. The last parameter informs on the database(s) to be queried for domain detection using InterProScan. RGAugury provides an optional parameter for specifying a companion Generic Feature Format (GFF3) or Gene Transfer Format (GTF) file when they are available. GFF3 or GTF files are helpful to draw RGA distribution plots using CViT [46] and draw gene and RGA domain structure plots. For most sequenced plant genomes, GFF3 annotation files can be downloaded from Phytozome [47].

Processing files derived from domain detection step and final RGA identification results are exported to plain text files. When a GFF file is provided, distribution plots of RGA families on chromosomes and the gene and domain structures for each RGA candidate are also generated.

### Implementation of the command-line version

A single command-line pipeline program was written in Perl to seamlessly process all analyses. As domain detection for genome-scale protein sequences is computationally intensive, performance was a critical factor considered during the design of RGAugury. Benefitting from the development of programming techniques and hardware, parallel computing can significantly boost large-scale data analysis. To parallel heavy analyses, a Perl module, *tool.pm*, which invokes the *fork* function to parallel the execution of BLAST searches in the initial RGA filtering and domain detection, was implemented.

### Web interface

**Fig. 3:**
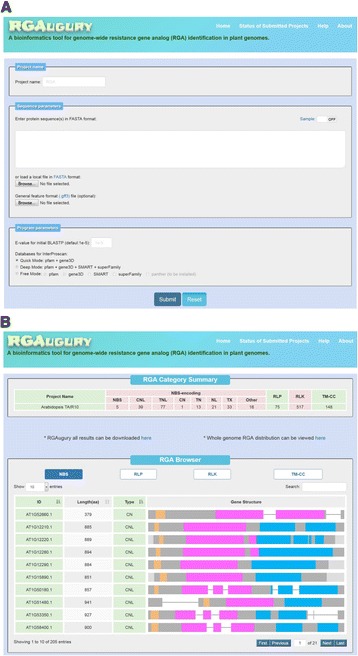
Web user interface pages of RGAugury. **a** The main page of RGAuguary for data input. All parameter values required in the command line version are specified directly on this page. Only protein sequences in FASTA format are required. A GFF3 file corresponding to the input protein sequences is optional but recommended. Databases for InterProscan can be selected by choosing either a predesigned ‘Quick’ mode or a ‘Deep’ mode. The default E-value cut-off for the initial RGA filtering with BLASTP is 1e-5. **b** The RGA prediction result summary page

To ease the command-line operations, a user-friendly web interface was designed and implemented using PHP and Java script (Fig. 3a). The necessary FASTA formatted protein sequences can be copied and pasted into the sequence text box or uploaded from a sequence file. A GFF3 file corresponding to the input protein sequences is optional but recommended. Databases for InterProScan can be selected through one of three modes: ‘Quick’, ‘Deep’ or ‘Free’ (flexible selection of up to five available databases). The default *P*-value cut-off for initial RGA filtering was set to 1e-5 but can be altered at user’s wish.

A project name is used as an intuitive ID for project management purposes. A ‘Job List Status’ page is dynamically generated upon project submission or following a mouse click on the ‘Status of Submitted Projects’ link (Additional file 2: Figure S1A). Finished jobs and current status of jobs being processed are displayed. Finished job are marked with the status “complete” in green that can be updated with the “Check” link to the ‘Results and Summaries’ page that shows the summary information, such as the numbers of RGAs and the detailed domain structure thumbnails for individual RGAs (Fig. 3b). The ‘Results and Summaries’ page also provides a link to download intermediate and final result files. All RGA candidates listed can be browsed. A search box is provided to query the candidates using full or partial letters or numbers from the table. For each RGA, an individual page will be generated by clicking on the Gene Structure Viewer (GSV) icon to show the details of the gene structure (Additional file 2: Figure S1B). GSV also provides links to the EnsemblePlants and GenBank websites for the genes that are available in these two databases, a useful feature for accessing additional gene information. When a GFF3 file is submitted along with the protein sequences, figures of RGA distribution on chromosomes are shown by clicking on the ‘Whole genome RGA distribution can be viewed’ link on Results and Summaries page (Additional file 2: Figure S1C).

The web version of RGAugury provides a new feature for InterProScan version check. When InterProScan runs on a web server, the web program will automatically check over the Internet whether the local InterProScan and its domain databases are up-to-date. If not, the web program pop-up dialog box reminding the user to update the software and its databases. In addition, detailed help pages are provided and synced with the RGAugury Bitbucket Wiki page (https://bitbucket.org/yaanlpc/rgaugury/wiki). Any updates on the Wiki page will also be reflected on the web help pages.

The RGAugury web program provides a project management function. Users can cancel executing jobs or delete finished projects from the server. Overall, the web version of RGAugury provides flexibility, convenience and interactive functionality over the command-line pipeline without compromising the RGA identification capabilities. The RGAugury web program can run on an HTTP server such as Apache. As hours of computing time are needed to finish a genome-scale RGA prediction, RGAugury is not suited for installation on a public web server. However, users are advised to download the pipeline and its web program from Bitbucket (https://bitbucket.org/yaanlpc/rgaugury) and install them in users’ local servers which can be used through intranet.

## Result and discussion

### RGA identification accuracy

Case studies were performed to test the prediction accuracy of the RGAugury pipeline. The protein sequences of the well annotated Arabidopsis genome (TAIR 10) [13, 25] were used for analysis. A total of 207 NBS-encoding genes were previously reported [27] but 14 of them were later considered rejected in TAIR10 as a consequence of erroneous annotations. The remaining 193 genes were used as a test data set for NBS-encoding genes. All protein sequences were downloaded from the NIBLRRS project website (http://niblrrs.ucdavis.edu). A total of 190 (98.5 %) out of 193 NBS-containing proteins were predicted and validated by the RGAugury pipeline (Table 1, Additional file 2). The three proteins with inconsistent prediction were AT5G45510, AT5G17950 and AT3G26470. AT5G45510 and AT5G17950 had an LRR domain but lacked an NB-ARC domain, while AT3G26470 was reported as *RPW8*, an oth-R type of resistance gene [48] that was presumed to have a TM and a CC domains rather than a NB-ARC domain [49]. As such, these three genes may have been originally incorrectly annotated [27]. If this inference is true, then the prediction accuracy of RGAugury is 100 % for NBS encoding genes in Arabidopsis. To further test RGAugury’s ability to detect genes harboring NBS motifs, all 27,416 unique Arabidopsis protein sequences (TAIR10) were processed through the pipeline. Besides the same 190 proteins mentioned above, an additional 15 NBS-encoding genes were predicted (Additional file 2). Of these newly predicted RGA genes, 6 NB-ARC domain containing genes were already annotated as disease resistance genes in TAIR10, the remaining 9 TX type genes were classified as unknown functions.

**Table 1:** Evaluation of RGA identification accuracy with RGAugury using the *Arabidopsis thaliana* dataset (TAIR10)

RGA type	No. of known RGAs	No. of RGAs identified	% identified
NBS	193	190	98.5
RLP	54	46	85.2
RLK	456	460	100.0

A similar evaluation was performed for RLP- and RLK-type RGAs. The previous report [50] indicated that the Arabidopsis genome encodes 57 RLPs, of which three have no sequence in TAIR 10, precluding their further analysis. Out of the remaining 54 RLPs, 46 (85.2 %) were predicted and 8 remained unclassified as a consequence of the absence of a TM domain (Table 1, Additional file 3). For RLKs, a total of 608 members were reported in the Arabidopsis genome, but only 75 % (456) of them were claimed to be associated with membranes [25]. RGAugury validated all 456 membrane-associated RLKs and identified additional four RLKs (Table 1, Additional file 4). The recently cloned wheat stem rust disease resistance genes *sr33* and *sr35* were also validated by RGAugury as NBS-LRR resistance genes [37, 38]. These case studies demonstrate that RGAugury accurately predicts RGAs on a genome-wide scale, and can be used for *R*-gene annotation.

### Computing performance and multiple thread optimization

RGAugury can run in either ‘Quick’ or ‘Deep’ mode which rely on the number of databases used for domain detection. Differences between the two modes resided mostly in the numbers of identified LRR containing proteins (Table 2). For example, LOC_Os11g42590.1, an NL type in rice, was identified as NBS type using the ‘Quick’ mode but as an NL type using the ‘Deep’ mode. The LRR domain in this gene can be identified by either Superfamily or SMART database through InterProScan. However, these differences were minor in most cases (Table 2). Thus, the ‘Quick’ mode is recommended for fast RGA annotation.

**Table 2:**
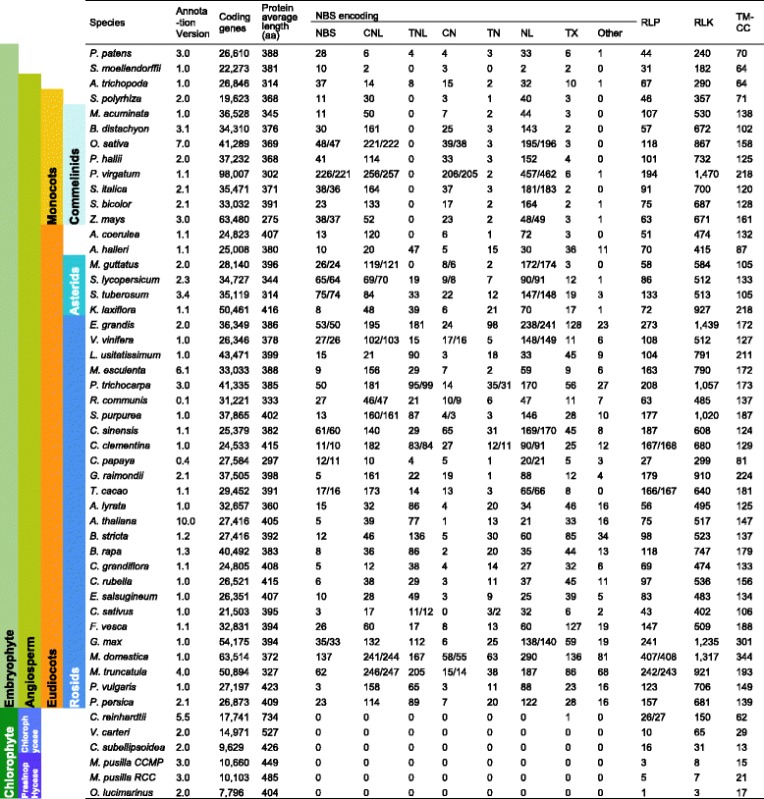
Summary of RGA identification results for 50 sequenced plant genomes

*Note*: Two modes for database selection were used: the ‘Quick’ mode (Pfam + Gene3D) and the ‘Deep’ mode (Pfam + Gene3D + SMART + Superfamily). Results were separated by a slash if differences existed between the two modes. Plants were sorted by taxonomic groups which are labelled on the left side of the table. ﻿*﻿A. coerulea*: *Aquilegia coerulea*; *A. halleri*: *Anemone halleri*; *A. lyrata*: *Arabidopsis lyrata*; *A. thaliana*: *Arabidopsis thaliana*; *A. trichopoda*: *Amborella trichopoda*; *B. distachyon*: *Brachypodium distachyon*; *B. rapa*: *Brassica rapa*; *B. stricta*: *Boechera stricta*; *C. clementina*: *Citrus clementina*; *C. grandiflora*: *Capsella grandiflora*; *C. papaya*: *Carica papaya*; *C. reinhardtii*: *Chlamydomonas reinhardtii*; *C. rubella*: *Capsella rubella*; *C. sativus*: *Cucumis sativus*; *C. sinensis*: *Citrus sinensis*; *C. subellipsoidea*: *Coccomyxa subellipsoidea*; *E. grandis*: *Eucalyptus grandis*; *E. salsugineum*: *Eutrema salsugineum*; *F. vesca*: *Fragaria vesca*; *G. max*: *Glycine max*; *G. raimondii*: *Gossypium raimondii*; *K. laxiflora*: *Kalanchoe laxiflora* ; *L. usitatissimum*: *Linum usitatissimum*; *M. acuminata*: *Musa acuminata*; *M. domestica*: *Malus domestica*; *M. esculenta*: *Manihot esculenta*; *M. guttatus*: *Mimulus guttatus*; *﻿M. pusilla*: *Micromonas pusilla*; *M. truncatula*: *Medicago truncatula*; *O. lucimarinus*: *Ostreococcus lucimarinus*; *O. sativa*: *Oryza sativa*; *P. hallii*: *Panicum hallii*; *P. patens*: *Physcomitrella patens*; *P. persica*: *Prunus persica*; *P. trichocarpa*: *Populus trichocarpa*; *P. virgatum*: *Panicum virgatum*; *P. vulgaris*: *Phaseolus vulgaris*; *R. communis*: *Ricinus communis*; *S. bicolor*: *Sorghum bicolor*; *S. italica*: *Setaria italica*; *S. lycopersicum*: *Solanum lycopersicum*; *S. moellendorffii*: *Selaginella moellendorffii*; *S. polyrhiza*: *Spirodela polyrhiza*; *S. purpurea*: *Salix purpurea*; *S. tuberosum*: *Solanum tuberosum*; *T. cacao*: *Theobroma cacao*; *V. carteri*: *Volvox carteri*; *V. vinifera*: *Vitis vinifera*; *Z. mays*: *Zea mays*

Though BLASTP has its own multiple thread parameter (*-n*), the paralleled tool, *tool.pm*, developed herein, outperformed it. Two threading methods were compared using 40 CPUs. BLASTP searches were performed for 5000 and 10,000 protein sequences randomly selected from the Arabidopsis genome against RGAdb using two threading methods (Additional file 5). An improvement of 3.8- and 3.4-fold in execution speed for the two datasets, respectively, was observed for the *tool.pm* module when compared to the thread parameter (*-n*) of BLASTP. Speed improvement of the domain detection programs nCoil, Phobius and pfam_scan theoretically depends on the called CPU numbers because they lack embedded multiple threads function. Thus, application of *tool.pm* to these programs significantly reduces the time needed for domain detection, making it possible to shorten computing time for genome-scale RGA identification to mere hours.

**Fig. 4:**
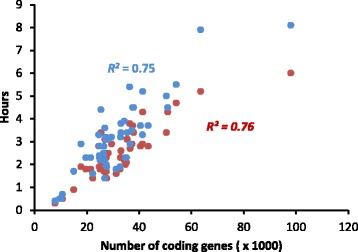
Performance of RGAugury. Forty-nine sequenced plant genomes (*Zea mays* was excluded, see text) with varying numbers of protein coding genes were used for RGA identification on a server embedded with 40 CPUs. Time to complete the processing of the entire pipeline for each dataset was recorded as a performance measurement. Performance for the ‘Quick’ mode (Pfam + Gene3D databases) and ‘Deep’ mode (Pfam + Gene3D + SMART + Superfamily) were compared. The dots and *R* ^*2*^ value in red represent results for the ‘Quick’ mode and those in red represent the results for the ‘Deep’ mode

To further evaluate the performance of RGAugury, we predicted RGAs for 50 sequenced plant genomes, including 44 angiosperm species and 6 green algae. All protein sequences were downloaded from the Phytozome (ver11.0) database website [47]. With both ‘Quick’ and ‘Deep’ modes, the processing time was highly correlated with the number of coding genes in genomes (*R* ^*2*^ = 0.76 for the ‘Quick’ mode and 0.75 for the ‘Deep’ mode) (Fig. 4). The total processing time averaged 2.4 h in ‘Quick’ mode. The ‘Deep’ mode averaged an extra 0.5 h but resulted in the enhanced prediction of LRR domains in some cases. Maize (*Zea mays*) data was not included in the correlation analysis because only 15.9 % of the input protein sequences were retained after the initial BLATP filtering compared with an average of 23.6 % among these 50 plant genomes, resulting in significantly fewer hours needed for the downstream domain identification. In general, RGAugury saves an average of 76 % time in the downstream domain/motif analysis if the initial BLASTP filtering step is performed before domain detection, demonstrating that the RGAugury pipeline is efficient for large-scale genome-wide RGA identification.

### Large-scale genome-wide RGA identification for comparative analysis

A summary of the RGA prediction results for the 50 sequenced plant genomes is shown in Table 2. NBS encoding genes are divided into two major groups based on their variable N-terminals: TIR and non-TIR [51]. It has been hypothesized that TNL have never evolved in monocots [52] or have been discarded during evolution [51, 53–55]. Tarr et al. [56] developed an indirect method to search for TNL in monocots and magnoliids based on degenerate PCR and, they did not observed any TNL in the studied monocots. Among the 50 genomes studied here, nine belong to three different monocot orders: Poales (*Brachypodium distachyon*, *Oryza sativa*, *Panicum hallii*, *Panicum virgatum*, *Setaria italic*, *Sorghum bicolor*, *Z. mays*), Zingiberales (*Musa acuminata*) and Alismatales (*Spirodela polyrhiza*). Our RGAugury prediction of these monocot species did not identify any TNL type confirming the previous findings (Table 2). Genes encoding TN or TX proteins were detected in these monocots but the numbers were smaller compared to genes predicted to encode other types of NBS motifs and compared to those from species other than monocots, implying that TNL may have been discarded during the evolution in the monocot lineage.

No genes coding for NB-ARC domains were predicted from the model green algae *Chlamydomonas reinhardtii*, *Volvox carteri*, and *Cyanidioschyzon merolae* because TIR and non-TIR NBS encoding genes arose only in the Plantae [57]. Our prediction results for all six green algae organisms confirmed this observation (Table 2), providing additional validation for the RGAugury prediction ability.

## Conclusions

We developed a command-line and a web version of an integrative, efficient and user-friendly pipeline called RGAugury, for large-scale genome-wide RGA prediction based on translated protein sequences. Four types of RGAs, namely NBS-encoding, RLP, RLK and TM-CC can be predicted. The results from validation data sets and the 50 sequenced plant genomes demonstrated its high accuracy and utility.

## Additional files

Additional file 1: Contents of the RGAdb database. (XLSX 31 kb)
Additional file 2: Figure S1. Additional web user interface pages of RGAugury. (A) The Status of Submitted Projects page. (B) Gene and domain structure page for an identified RGA. Detailed information is included in a spreadsheet result file. External links to NCBI and EnsemblPlants are indicated with their respective website logo. (C) RGA distribution on chromosomes. NBS encoding, RLP, RLK and TM-CC RGAs are represented by different color bars. ALL represents a combined RGA distribution figure for the merged data of all four RGA families. (PPTX 359 kb)
Additional file 3: Prediction results for NBS-encoding genes in the Arabidopsis genome. (XLSX 58 kb)
Additional file 4: Prediction results for RLP genes in the Arabidopsis genome. (XLSX 11 kb)
Additional file 5: Prediction results for RLK genes in the Arabidopsis genome. (XLSX 21 kb)

## Abbreviations

CC: Coiled-coil
CN: CC-NBS
CNL: CC-NBS-LRR
ETI: Effector-triggered immunity
GFF: Generic feature format
GPL: GNU general public license
GSV: Gene structure viewer
GTF: Gene transfer format
LRR: Leucine rich repeat
LysM: Lysin motif
LZ: Leucine zipper
NB-ARC: Nucleotide binding adaptor shared by APAF-1, certain *R* gene products and CED-4
NBS: Nucleotide-binding site
NL: NBS-LRR
PAMP/MAMP: Pathogen/microbe-associated molecule pattern
PRR: Pattern-recognition receptor
PTI: PAMP triggered immunity
RGA: Resistance gene analog
RLK: Receptor like kinase
RLP: Receptor like protein
STK: Serine-threonine kinase
STTK: Serine/threonine and tyrosine kinase
TIR: Toll/Interleukin-1 receptor
TM: Transmembrane
TN: TIR-NBS
TNL: TIR-NBS-LRR
TX: TIR-unknown domain.
