# Supplementary figures and images for: RGAugury: a pipeline for genome-wide prediction of resistance gene analogs (RGAs) in plants
Source: BMC Genomics. 2016 Nov 2;17:852. doi: 10.1186/s12864-016-3197-x (PMC5093994; doi:10.1186/s12864-016-3197-x)

## Slide 1
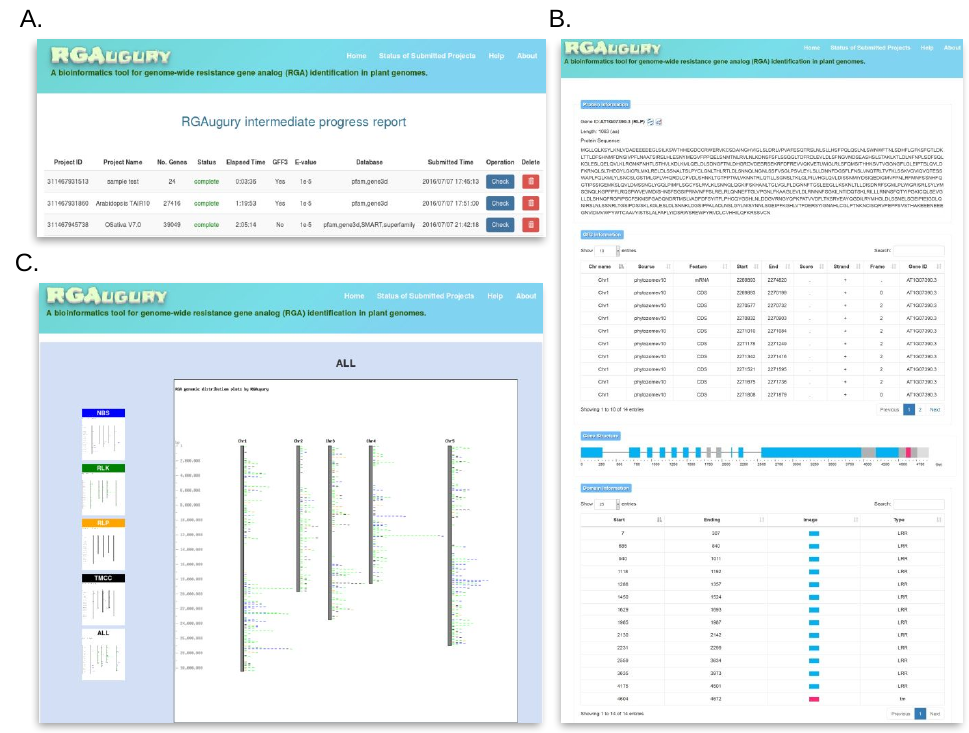

A.
B.
C.

Supplement: Additional file 2: Figure S1. — Additional web user interface pages of RGAugury. (A) The Status of Submitted Projects page. (B) Gene and domain structure page for an identified RGA. Detailed information is included in a spreadsheet result file. External links to NCBI and EnsemblPlants are indicated with their respective website logo. (C) RGA distribution on chromosomes. NBS encoding, RLP, RLK and TM-CC RGAs are represented by different color bars. ALL represents a combined RGA distribution figure for the merged data of all four RGA families. (PPTX 359 kb) [file 12864_2016_3197_MOESM2_ESM.pptx]
